# Evaluation of the biofilm life cycle between *Candida albicans* and *Candida tropicalis*

**DOI:** 10.3389/fcimb.2022.953168

**Published:** 2022-08-18

**Authors:** María Belén Atiencia-Carrera, Fausto Sebastián Cabezas-Mera, Karla Vizuete, Alexis Debut, Eduardo Tejera, António Machado

**Affiliations:** ^1^ Universidad San Francisco de Quito (USFQ), Colegio de Ciencias Biológicas y Ambientales COCIBA, Instituto de Microbiología, Laboratorio de Bacteriología, Quito, Ecuador; ^2^ Center of Nanoscience and Nanotechnology, Universidad de las Fuerzas Armadas (ESPE), Sangolquí, Ecuador; ^3^ Facultad de Ingeniería y Ciencias Agropecuarias Aplicadas, Grupo de Bioquimioinformática, Universidad de Las Américas (UDLA), Quito, Ecuador

**Keywords:** biofilms, life cycle, *Candida albicans*, *Candida tropicalis*, epifluorescence microscopy, scanning electron microscopy

## Abstract

*Candida tropicalis* is an emergent pathogen with a high rate of mortality associated with its biofilm formation. Biofilm formation has important repercussions on the public health system. However, little is still known about its biofilm life cycle. The present study analyzed the biofilm life cycle of *Candida albicans* and *C. tropicalis* during various timepoints (24, 48, 72, and 96 h) through biomass assays, colony-forming unit (CFU) counting, and epifluorescence and scanning electron microscopies. Our results showed a significant difference between *C. albicans* and *C. tropicalis* biofilms in each biomass and viability assay. All-time samples in the biomass and viability assays confirmed statistical differences between the *Candida* species through pairwise Wilcoxon tests (*p* < 0.05). *C. albicans* demonstrated a lower biomass growth but reached nearly the same level of *C. tropicalis* biomass at 96 h, while the CFU counting assays exhibited a superior number of viable cells within the *C. tropicalis* biofilm. Statistical differences were also found between *C. albicans* and *C. tropicalis* biofilms from 48- and 72-h microscopies, demonstrating *C. tropicalis* with a higher number of total cells within biofilms and *C. albicans* cells with a superior cell area and higher matrix production. Therefore, the present study proved the higher biofilm production of *C. tropicalis*.

## 1 Introduction

*Candida* species are widely distributed in nature, normally as a part of commensal mammalian microbiota (Graf et al., 2019). However, alterations in the host environment, including disruptions in commensal microbiota, might trigger the transition from the commensal to a pathogenic phase (Alves et al., 2020). In the last decades, fungal infections in humans are becoming an emergent problem in the public health system and are considered by many authors as a neglected infectious disease (Bassetti et al., 2019; Kobayashi et al., 2020; Rodrigues and Nosanchuk, 2020). Nowadays, more than 200 species of *Candida* have been described (Pohl, 2022). Although *Candida albicans* remains the most prevalent fungal pathogen, the morbidity and mortality caused by non-albicans *Candida* (NAC) species are increasing (Dhale et al., 2014; Zhang et al., 2020). Besides *C. albicans*, there are four emerging NAC species, more specifically, *Candida tropicalis*, *Candida parapsilosis*, *Candida glabrata*, and *Candida krusei* (Atiencia-Carrera et al., 2022). Among these, *C. tropicalis* is now considered the most important emerging fungal pathogen, and recent reports have identified several strains resistant to standard empirical treatments, such as fluconazole (Tsay et al., 2020; Atiencia-Carrera et al., 2022).

Both *C. albicans* and *C. tropicalis* are known to possess a broad range of virulence factors and commensal characteristics conferring the ability to colonize and invade host tissue (de Barros et al., 2020). These factors include the expression of adhesins and invasins on the cell surface, ability to damage host cells, thigmotropism (contact sensing), phenotypic switching, secretion of hydrolytic enzymes, and formation of biofilms (de Barros et al., 2020). Although it is well known that biofilms represent the most prevalent growth form of microorganisms (Nobile and Johnson, 2015; Flores-Vargas et al., 2021) and that biofilm formation among *Candida* species confers significant resistance to antifungal therapy (Alves et al., 2020; Atriwal et al., 2021), little is still known about the biofilm life cycle of *C. tropicalis*. In 2017, Kawai and colleagues (Kawai et al., 2017) evaluated the *C. tropicalis* biofilm formation and the antifungal efficacy of liposomal amphotericin B using time-lapse imaging, showing *C. tropicalis* as a fast-growing type and able to form aggressive biofilms. However, Kawai and colleagues only analyzed *C. tropicalis* biofilms for 24 h (Kawai et al., 2017).

The ability to establish a biofilm is considered a main virulence factor among pathogens, limiting the penetration of substances through the matrix and protecting cells from host immune responses (Soll and Daniels, 2016; Hall and Mah, 2017). In addition, mature biofilms can evade the host immune system (Alves et al., 2020; Eix and Nett, 2020). It is assumed that the formation of mature biofilms and subsequent production of extracellular matrix is strongly dependent on the species, strain, and environmental conditions (such as pH, medium composition, and oxygen, among others) (Silva et al., 2017; Atriwal et al., 2021). Therefore, it is important to evaluate the biofilm life cycle among pathogens like *C. albicans* and *C. tropicalis*.

Our recent meta-analysis on the prevalence of *Candida* biofilms in bloodstream infections showed that 70.0% of the mortality rate was from biofilm-associated infections (Atiencia-Carrera et al., 2022), evidencing *C. tropicalis* as the prevalent species. Although several authors reported *C. tropicalis*’ ability to establish a strong biofilm in the last two decades (Al-Fattani and Douglas, 2006; Mohandas and Ballal, 2011; Banerjee et al., 2015; Weerasekera et al., 2016; Marak and Dhanashree, 2018; Sahal and Bilkay, 2018), no meticulous evaluation was carried out on the life cycle of this species over 96 h through the four classical methodologies normally used for biofilm analysis, so the present work aimed to compare the biofilm cycle of life between *C. albicans* and *C. tropicalis* and characterize their biofilm production through *in vitro* conditions. This study analyzed the biofilms of these *Candida* species during the time window of 24, 48, 72, and 96 h by biomass growth assays [optical density measurement at 630 nm using crystal violet staining and phosphate-buffered saline (PBS) suspension], colony-forming unit (CFU) counting, epifluorescence microscopy (EM), and scanning electron microscopy (SEM).

## 2 Materials and methods

### 2.1 Fungal isolates and growth conditions

Two *Candida* species, *C. albicans* of the American Type Culture Collection ATCC^®^ 10231™ (American Type Culture Collection, ATCC) and *C. tropicalis* isolate from the microbial collection of the Institute of Microbiology Universidad San Francisco de Quito (designated as IMUSFQ-V546), were selected for the present study. *C. tropicalis* isolate IMUSFQ-V546 was previously recovered from a patient with invasive candidiasis and identified through DNA sequences at multiple loci and biochemical properties in the National Institute for Research in Public Health (INSPI, 2021). Strains were stored at -80 or -20°C, and 24 h before each assay, a new culture in Sabouraud dextrose agar (SDA; Dipco Cía. Ltda., Quito, Ecuador) was made to avoid natural mutants (Soll and Daniels, 2016). After their growth for 24 h at 37°C, yeast cells were harvested and suspended in PBS to obtain the cellular density using a UV–vis spectrometer (GENESYS™ 20 Thermo Scientific™, Waltham, Massachusetts, USA). At 540 nm, the cellular density was adjusted at 1 × 10^7^ CFU per milliliter. The cellular density was obtained with the growth curve of the two strains (Chandra et al., 2008; Silva et al., 2009) (see the **Supplementary Material**).

### 2.2 Biofilm formation

The appropriate inoculum in PBS was centrifugated at 400 rpm for 10 min, and the pellet was resuspended in sterile Sabouraud dextrose broth (Dipco Cía. Ltda., Quito, Ecuador). In each well of the six-well plate containing a sterile coverslip, 3 ml of primary biofilm inoculum (1 × 10^7^ CFU per ml) was added. Also, blank control was prepared in the same plate, which also contained a cover slip but placed in a sterile medium (Chandra et al., 2008). The plates were incubated at 37°C for different periods (24, 48, 72, and 96 h) under static conditions, replacing the previous medium in each well with 3 ml of fresh medium every 24 h after the biofilm samples were washed with PBS (Lohse et al., 2018). Each assay was performed with at least three replicates per strain and growth period. In each replicate assay, we also prepared two samples of biofilm by strain to perform the biomass assays separately.

### 2.3 Biomass quantification

#### 2.3.1 Crystal violet staining

After a certain period of growth (24, 48, 72, and 96 h), the biofilm samples were carefully washed with 3 ml of sterile PBS. Then, the coverslips containing the biofilm sample were transferred to a clean six-well plate and stained with 3 ml of crystal violet (CV, 1% v/v) for 45 min, and the excess stain was carefully removed from the wells. Furthermore, 3 ml of alcohol 96% (v/v) was placed into each well for 5 min, and finally, 200 μl of the biofilm sample was placed in a 96-well plate and read in the ELISA Elx808 spectrophotometer (BioTek, Winooski, USA) at 630 nm. All biofilm samples, the blank controls, and also a well with pure alcohol were included in the 96-well plate (Gulati et al., 2018).

#### 2.3.2 Phosphate-buffered saline suspension

After the biofilm formation assays, the second set of biofilm samples was also carefully washed with 3 ml of sterile PBS. Then, each coverslip containing the biofilm sample was placed in a sterile plastic flask with 3 ml of sterile PBS and vortexed at maximum velocity for 5 min to ensure the biofilm remotion off the coverslip and into the PBS solution (Gulati et al., 2018). For each sample, 200 μl of the previous suspension was placed in a 96-well plate and read in the ELISA Elx808 spectrophotometer at 630 nm. All biofilm samples, the blank controls, and sterile PBS were likewise measured (Gulati et al., 2018). The remaining PBS suspension was used for the viability quantification assays.

### 2.4 Viability quantification

#### 2.4.1 Colony-forming unit counting

To enumerate culturable sessile cells, a CFU counting assay was used. At least five individual PBS suspensions of each biofilm sample were used in a serial 10-fold dilution by adding 100 μl of sample to 900 μl of sterile PBS. Each dilution was thoroughly vortexed, and the pipette tips were changed before the next dilution or experimental step. Dilutions of 10^-3^, 10^-4^, and 10^-5^ were plated on SDA by triplicate, resulting in 15 plates per biofilm sample. The plates were incubated for 24 h at 37°C, after which the colonies were counted (Nailis et al., 2010). The experiments were performed at the same time as the biomass experiments; thus, three CFU assays per dilution were available for analysis, and data were collected. For statistical analysis, the dilution with a growth between 25 and 250 CFU was chosen according to previous studies (Merritt et al., 2005; Thomas et al., 2015).

#### 2.4.2 Fluorescence staining

After the evaluation of CFU counting and biomass quantification assays, 48- and 72-h time samples were chosen to be analyzed by fluorescence staining. After each biofilm formation in six-well plates, any remaining medium in the wells was removed, and three coverslips were transferred to a fresh six-well plate. A working solution of fluorescent stains was prepared by adding 10  μl of SYTO^®^ 9 stain and 10  μl of propidium iodide (PI) stain (FilmTracer™ LIVE/DEAD^®^ Biofilm Viability Kit, Invitrogen, Carlsbad, CA, USA) to 10  ml of filter-sterilized water in a foil-covered container (dead–alive working solution). In addition, another working solution was prepared using 20  μl of 4′,6-diamidino-2-phenylindole stain (DAPI, Sigma Aldrich #10236276001, St. Louis, Missouri, USA) in 10  ml of filter-sterilized water in a foil-covered container (DAPI working solution). These two working solutions were stored at -20°C. About 200  μl of the live/dead working solution was added onto each coverslip (biofilm sample) gently so as not to disturb the biofilm. The samples were incubated for 30 min at room temperature and protected from light before being rinsed with 200 µl of PBS. Then, 200  μl of DAPI working solution was also added to the previous biofilm sample and incubated for 10 min at room temperature, protected from light, and finally washed again with 200 µl of PBS. Each coverslip was then placed face up onto a clean, dry microscope slide, and a drop of mounting medium was added (ProLong Gold Antifade, ThermoFisher Scientific, MA, USA). An autoclavable 22-mm-diameter glass coverslip (Dipco Cía. Ltda., Quito, Ecuador) was used to fix the sample in place. The samples were stored protected from light at room temperature (25°C) until epifluorescence microscopy was performed within the first hour (Rosenberg et al., 2019; Mountcastle et al., 2021).

### 2.5 Epifluorescence microscopy

EM was carried out using an Olympus BX50 microscope (Olympus Corporation, Tokyo, Japan) equipped with a ×100 oil immersion objective. Images were captured with AmScope Digital Camera MU633-FL (AmScope, California, USA) and digitalized with AmScope software version 1.2.2.10. As previously described by Rosenberg and colleagues (Rosenberg et al., 2019), for counting purposes, at least 12 images were taken per sample on the 22-mm-diameter glass coverslip at random locations. For a more reproducible result presentation, cell/yeast counts are given per square centimeter. The number of *Candida* cells was counted from each field to obtain the average number of cells over the total area of ​​the abiotic surface. Briefly, the coverslip area (4.84E + 08 μm^2^) was divided by the area of ​​the picture (12,880 μm^2^) and the average of cells from microscopic fields was multiplied by the previous ratio, thus obtaining the total number of cells over the abiotic glass surface. These results were expressed as the number of cells ± standard deviation per square centimeter (*N* cells/cm^2^ ± SD) by dividing the previous total number of cells and their deviation over the glass surface area in square centimeter (4.84 cm^2^). In EM, the percentages of dead and alive cells within images were measured through ImageJ by Fiji (Schindelin et al., 2012) (version 1.57) using the macros Biofilms Viability checker proposed by Mountcastle et al. (2021) and the plugin MorphoLibJ (Legland et al., 2016), while the total cell counting in DAPI images was processed by a sequence of modules forming a pipeline designed for this purpose in Cell Profiler software (Mcquin et al., 2018), an open-source software, version 4.2.1 (available from the Broad Institute at www.cellprofiler.org), the applied pipeline of which can be reviewed in the **Supplementary Material**. The DAPI images were used to obtain the total number of cells per image, and the average was then calculated as the mean of yeasts per square centimeter.

### 2.6 Scanning electron microscopy

Samples grown for 48 and 72 h were also selected to be examined by SEM. For the SEM analysis, 22-mm circular cover glasses (Heathrow Scientific, Vernon Hills, Illinois, USA) were placed in a six-well plate, and *Candida*-related biofilms were formed as previously described. The pre-formed biofilms were fixed by adding in the wells a solution of PBS concentration adjusted to pH = 4.7 containing glutaraldehyde at 4% for 1 h. Post-fixation was carried out with 1% osmium tetroxide in cacodylate buffer for 1 h. Subsequently, the samples were treated with 1% tannic acid for 1 h. The samples were dehydrated with a series of ethanol washes of 30 min each, with the solutions containing 30, 50, 70, 80, 90, and 100% of ethanol in distilled water; the samples were further dried with CO_2_ in a critical point dryer (Balzers CPD 030, Schalksmühle, Germany) (Melo et al., 2011; Marcos-Zambrano et al., 2014). Finally, discs with biofilm were coated with gold, and the morphological analysis was elaborated using a Tescan Mira 3 scanning electron microscope equipped with a Schottky Field Emission Gun (Schottky FEG-SEM, MIRA III TESCAN, Brno, Czech Republic) at the Centro de Nanociencia y Nanotecnología of the Universidad de las Fuerzas Armadas ESPE (Pilaquinga et al., 2019). The morphology of the yeast was also obtained from the best images by Fiji ImageJ (Schindelin et al., 2012) (version 1.57), in which the mean yeast area (µm^2^) was measured through the average area of *Candida* cells obtained by each picture from triplicate assays in the SEM analysis.

### 2.7 Statistical analysis

All data of the present study were obtained from at least triplicate assays performed on different days. In the case of biomass growth and CFU counting assays, each assay was performed with five replicates. In addition, the raw results from biomass growth assays were subtracted by the negative OD control values. Then, the standard deviation (SD) was determined for each data set of the results. We assessed the data distribution using the Shapiro–Wilk test. If the data had a normal distribution, we used parametric hypothesis test to compare two or more samples. If the data had a non-normal distribution, non-parametric tests were used. Medians between times in the same *Candida* species were compared by using the Kruskal–Wallis nonparametric test, followed by Dunn’s test using a Benjamini–Hochberg adjustment for multiple comparisons test at *α* = 0.05, except between two-time samples where pairwise Wilcoxon test was applied—more specifically, the EM and SEM analyses. Meanwhile, between *Candida* species (interspecies), the results were compared using pairwise Wilcoxon test. Least-squares linear regression models were used to compare the four methods to assess biofilm development—more specifically biomass quantification by CV staining and PBS suspension, total cell count by EM analysis, and CFU counting assays. All data analyses were performed in R studio version 4.0 (RStudio Team, 2021) using several R packages (“ggpubr”, “rstatixs”, “openxlsx”, and the “tidyverse” set of packages) (Wickham et al., 2019; Kassambara, 2021). Finally, all *p-*values <0.05 were considered significant.

## 3 Results

### 3.1 Quantification of the *C. albicans* and *C. tropicalis* biofilms and their normality assessment

The ability to develop a biofilm by *C. albicans* ATCC^®^ 10231™ and *C. tropicalis* V453 was determined by comparing biomass against viability (**Table 1**). A normality assessment, through the Shapiro–Wilk test and histogram examination, was also applied to the obtained data (see **Supplementary Material Information S1**).

**Table 1:** Summary of the results and statistical analysis obtained from the biomass and viability assays of growth with *Candida albicans* and *Candida tropicalis*.

Timepoints	Variable	*Candida albicans*			*Candida tropicalis*			Normality assessment	
		Total samples^a^	Mean	Median	Total samples^a^	Mean	Median	Shapiro–Wilk test^b^	
			(SD)	(min–max)		(SD)	(min–max)	Statistics	*P*-value
**24h**	Biomass PBS A_630_	20	0.280	0.277	20	0.667	0.702	0.9521432	8.99E - 02
			(0.127)	(0.101–0.596)		(0.160)	(0.362–0.952)		
	Biomass CV A_630_	20	0.056	0.058	20	0.151	0.146	0.8819685	5.95E - 04
			(0.014)	(0.023–0.077)		(0.018)	(0.117–0.185)		
	Viability CFU/ml	20	1.22E + 08	1.13E + 08	20	9.89E + 08	1.03E + 09	0.8163214	1.51E - 05
			(2.23E + 07)	(8.00E + 07–1.93E + 08)		(1.60E + 08)	(5.30E + 08–1.39E + 09)		
**48h**	Biomass PBS A_630_	15	0.562	0.539	20	0.936	0.968	0.9457164	8.36E - 02
			(0.184)	(0.271–0.928)		(0.126)	(0.708–11.455)		
	Biomass CV A_630_	15	0.168	0.175	20	0.270	0.270	0.8670426	5.76E - 05
			(0.015)	(0.145–0.194)		(0.026)	(0.194–0.303)		
	Viability CFU/ml	15	2.49E + 08	2.30E + 08	20	2.33E + 09	2.12E + 09	0.8419846	1.60E - 04
			(4.59E + 07)	(1.33E + 08–4.67E + 08)		(2.79E + 08)	(1.80E + 09–3.47E + 09)		
**72h**	Biomass PBS A_630_	15	0.822	0.819	15	1.170	1.184	0.6378376	2.18E - 11
			(0.169)	(0.548–1.116)		(0.090)	(0.919–1.131)		
	Biomass CV A_630_	15	0.269	0.269	15	0.328	0.333	0.9499481	1.68E - 01
			(0.011)	(0.221–0.291)		(0.038)	(0.257–0.384)		
	Viability CFU/ml	15	4.13E + 08	3.93E + 08	15	2.99E + 09	2.93E + 09	0.8090880	9.64E - 05
			(1.09E + 08)	(2.10E + 08–5.93E + 08)		(3.09E + 08)	(2.07E + 09–4.13E + 09)		
**96h**	Biomass PBS A_630_	15	1.045	1.033	15	1.290	1.419	0.251759	4.81E - 11
			(0.109)	(0.885–1.306)		(0.130)	(1.085–1.141)		
	Biomass CV A_630_	15	0.314	0.318	15	0.338	0.347	0.9697337	5.32E - 01
			(0.031)	(0.264–0.369)		(0.045)	(0.223–0.423)		
	Viability CFU/ml	15	4.77E + 08	4.67E + 08	15	5.58E + 09	6.00E + 09	0.7540960	1.06E - 05
			(3.05E + 07)	(4.23E + 08–5.83E + 08)		(8.22E + 08)	(3.87E + 09–6.80E + 09)		

Evaluation of biofilm life cycle of two Candida species *in vitro* assays, where the values of the biomass assays are shown without the absorbance values obtained in the negative controls of each assay.

a At least five samples per assay and each assay was realized in triplicate on three different days.

b Shapiro–Wilk tests evaluated the data distribution; more specifically, if the p-value is equal to or less than 0.05, it is a non-normal distribution and non-parametric statistical analysis must be performed.

As shown in **Table 1**, *C. tropicalis* biofilms had a higher capacity to produce biomass and colony-forming unit counting *in vitro* during all time samples when compared to *C. albicans* biofilms. The Shapiro–Wilk tests evidenced a non-normal distribution, showing *p*-values <0.05 apart from two sample times in CV assays (72 and 96 h) and one sample time in PBS assays (24 h). Therefore, most of the data showed a non-normal distribution among the results, and consequently, a non-parametrical statistical analysis was selected for future evaluation.

### 3.2 Evaluation of the intraspecies biofilm growth

**Figure 1:**
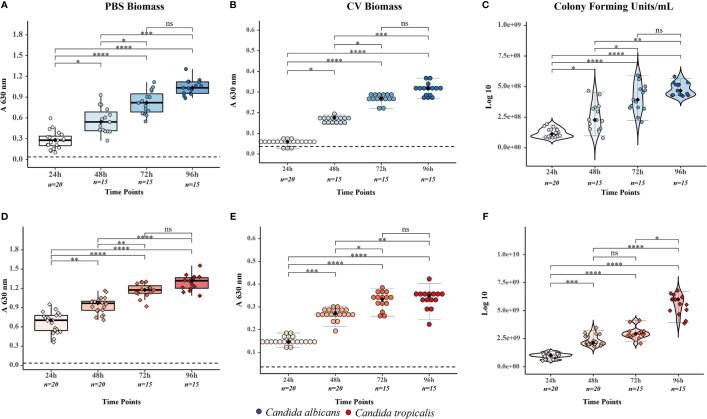
Evaluation of the biofilms formed by *Candida albicans* and *Candida tropicalis* during the specified time window (24, 48, 72, and 96 h) through the biomass and viability assays. Biofilm biomass was evaluated through two methodologies of optical density measurement, while viable cells within the biofilm were analyzed through colony-forming unit counting to describe the number of viable microorganisms. All these results were grouped and plotted for each species, where color dots represent the individual result of each assay (blue dots for (C) albicans and red dots for (C) tropicalis), overlaid box plots cover the upper and lower interquartile ranges, whiskers extend to extreme datapoints, and the black diamond represents the median (data shown in **Table 1**). **(A)** Biomass of (C) albicans biofilm by phosphate-buffered saline (PBS) suspension method, **(B)** biomass of (C) albicans biofilm by crystal violet (CV) staining method, **(C)** viability of (C) albicans biofilm by colony-forming unit counting, **(D)** biomass of (C) tropicalis biofilm by PBS suspension method, **(E)** biomass of (C) tropicalis biofilm by CV staining method, and **(F)** viability of (C) tropicalis biofilm by colony-forming unit counting. The illustrated statistical analysis between time in biomass and viability assays for each Candida species was realized through post-hoc Dunn’s test using a Benjamini–Hochberg adjustment (all data are shown in **Supplementary Material Information S1**), more specifically *p < 0.05; **p < 0.01; ***p < 0.001; ****p < 0.0001; ns, non significant.

Biofilm biomass and viable cells within the biofilm were quantified and further analyzed for intraspecies statistical differences during the specified time window (**Figure 1**). The Kruskal–Wallis test demonstrated a significant effect between time samples in the biomass growth and viability of biofilms in both *Candida* species (*p* < 0.0001; see **Supplementary Material Information S1**).

The *C. albicans* biofilms had a significant increase in biomass and number of viable cells up to 72 h of growth, with no difference between 72 and 96 h. The *C. tropicalis* biofilms had a significant increase in the number of viable cells from 24 to 48 h, but not from 48 to 72 h. However, the viable cells increased in number from 72 to 96 h. It is worth mentioning that the increment in viable cells within the *C. tropicalis* biofilm during this period was surprisingly superior to the previous periods despite the stationary biomass growth in *C. tropicalis* biofilm.

### 3.3 Evaluation of the interspecies biofilm growth

The overall results evidenced a significant difference between *C. albicans* and *C. tropicalis* biofilms in each of the biomass and viability assay. All-time samples in the biomass growth and viability assays likewise confirmed statistical differences between *Candida* species through multiple pairwise comparisons with Wilcoxon tests (**Table 2**). Interestingly, it is possible to observe that both PBS and CV assays showed the same biomass growth tendency, where *C. albicans* demonstrated a lower biomass growth but reached nearly the same level to that of *C. tropicalis* biomass at 96 h. However, the CFU counting assays did not show the same pattern and constantly demonstrated a higher number of viable cells within the *C. tropicalis* biofilm (approximately a CFU counting difference of around 1.00E + 01 CFU/ml in all-time samples).

**Table 2:** Evaluation of the statistical differences in biofilm growth between *Candida albicans* and *Candida tropicalis* through biomass and viability assays.

Biofilm growth	*C. albicans*			*C. tropicalis*			Non-parametrical statistical analysis (interspecies)	
Assays	*n* ^a^	Mean	SD	*n* ^a^	Mean	SD	Wilcoxon test *p*-value	
Biomass PBS A_630_
24 h	4	0.280	0.127	4	0.667	0.160	4.81E - 07	
48 h	3	0.562	0.184	3	0.936	0.126	1.16E - 05	
72 h	3	0.822	0.169	3	1.170	0.090	1.44E - 05	
96 h	3	1.045	0.109	3	1.290	0.130	3.99E - 05	
Biomass CV A_630_
24 h	4	0.056	0.014	4	0.151	0.018	5.44E - 08	
48 h	3	0.168	0.015	3	0.270	0.026	5.54E - 07	
72 h	3	0.269	0.011	3	0.328	0.038	7.00E - 04	
96 h	3	0.314	0.031	3	0.338	0.045	4.33E - 02	
Viability CFU/ml
24 h	4	1.22E + 08	2.23E + 07	4	9.89E + 08	1.60E + 08	6.67E - 08	
48 h	3	2.49E + 08	4.59E + 07	3	2.33E + 09	2.79E + 08	6.17E - 07	
72 h	3	4.13E + 08	1.09E + 08	3	2.99E + 09	3.09E + 08	3.37E - 06	
96 h	3	4.77E + 08	3.05E + 07	3	5.58E + 09	8.22E + 08	3.27E - 06	

The standard deviation (SD) and the mean were calculated by the average values of the total number of assays (five samples per assay). The data set showed a non-normal distribution among the results, and consequently, a non-parametrical statistical analysis was selected to evaluate statistical differences in biofilm growth between *C. albicans* and *C. tropicalis*. More specifically, the results between Candida species were evaluated using the Wilcoxon test. ^a^Number of assays realized on different days for each time sample.

### 3.4 Live/dead cells and cell morphologies of the *C. albicans* and *C. tropicalis* biofilms

Next, we decided to analyze the amount of live/dead cells and cell morphologies within biofilms between *Candida* species during the exponential phase and initial stationary phase. As shown in **Table 3**, the EM analysis evidenced a significant effect between samples according to time of growth in both *Candida* species. However, the SEM analysis showed no statistical differences in the yeast cell areas between 48 and 72 h in both *Candida* species. When comparing species, statistical differences were observed in 48- and 72-h biofilms from *C. albicans* and *C. tropicalis* in both methods. *C. tropicalis* demonstrated a higher number of total cells within biofilms at 48 and 72 h when compared to *C. albicans* biofilms and as expected from previous CFU counting assays. However, no statistical differences were found in the percentage of dead and alive cells within the biofilm between these *Candida* species. Interestingly, the mean size of *C. albicans* cell area at 48 and 72 h was statistically superior when compared to that of *C. tropicalis* cells in both time samples, although a decrease in cell area was observed during the stationary phase.

Table 3Evaluation of the total cell counts, live/dead cells and cell morphologies between biofilms of *Candida albicans* and *Candida tropicalis* at 48 and 72h.

**Table:** 

Epifluorescence microscopy (EM) with DAPI Staining and LIVE/DEAD Biofilm Viability Kit													
* *	*C. albicans*						*C. tropicalis*						Non-parametrical statistical analysis Interspecies
Assays	n ^a^	Mean of yeasts/frame ^b^ (SD)	Mean of yeasts/cm^2 c^ (SD)	Dead(SD) %	Alive (SD) %	Wilcoxon test *p*-value	Mean of yeasts/frame(SD)	Mean of yeasts/cm^2^ (SD)	Dead(SD) %	Alive (SD) %	Wilcoxon test *p*-value		Wilcoxon test*p*-value
48h	3	1.56E+03 (2.94E+02)	1.20E+07 (2.25E+06)	5.00 (0.53)	95.00 (1.00)	1.29E-08	2.18E+04 (1.41E+03)	1.67E+08 (1.09E+07)	4.30 (0.50)	95.70 (0.20)	6.19E-10		6.17E-10
72h	3	5.55E+04 (1.18E+04)	4.27E+08 (9.07E+07)	17.00 (3.18)	83.00 (3.00)		8.03E+04 (6.61E+03)	6.19E+08 (5.11E+07)	17.40 (4.50)	82.60 (5.00)			5.04E-08

**Table:** 

Scanning Electron Microscopy (SEM) with Morphology of yeasts								
* *	*C. albicans*			* *	*C. tropicalis*			Non-parametrical statistical analysis Inter-species
Assays	n ^a^	Mean size of the yeast cell area, µm^2^ (SD) ^d^	Wilcoxon test *p*-value		Mean size of the yeast cell area, µm^2^ (SD) ^d^	Wilcoxon test *p*-value		Wilcoxon test *p*-value
48h	3	1.80E-02 (3.00E-03)	2.22E-01		1.00E-02 (2.00E-03)	4.44E-01		3.05E-05
72h	3	1.60E-02 (2.00E-03)			1.10E-02 (2.00E-03)			3.00E-05

48 and 72h time samples were selected to compare the total cell counts, number of live/ dead cells, as well as biofilm structure and cell area for each Candida species using epifluorescence microscopy (EM) and scanning electron microscopy (SEM) analysis, respectively. In EM, the percentages of dead and alive cells within images were measured through ImageJ by Fiji version 1.57 (Schindelin et al., 2012) using the macros Biofilms Viability checker (see methods); while, the total cells counting in DAPI images were processed by a sequence of modules forming a pipeline in Cell Profiler software (Mcquin et al., 2018), which the applied pipeline can be revised in the **Supplemental Material**. DAPI images were used to obtain total cells per image and the average was then calculated as the mean of yeasts per cm^2^. Morphology of the yeast was also obtained from the best images by Fiji ImageJ version 1.57 (Schindelin et al., 2012). Wilcoxon test was applied to evaluate statistical differences between 48 and 72h samples in each *Candida* species (Intraspecies). In non-parametrical statistical analysis (Interspecies), the Wilcoxon test was calculated using the results between *C. albicans* and *C. tropicalis* at 48 and 7h samples. ^a^Number of assays realized on different days; in each assay, we collected at least 12 photographs for cell counting. ^b^Average of Candida cells obtained by pictures from triplicate assays and their standard deviation (SD). ^c^The estimation of yeast/cm2 calculated by the following formula: average of *Candida cells* (SD)* (1E+08/12880). ^d^The mean of yeast area (µm^2^) measured through the average area of Candida cells obtained by each picture in SEM analysis and their standard deviation (SD).

**Figure 2:**
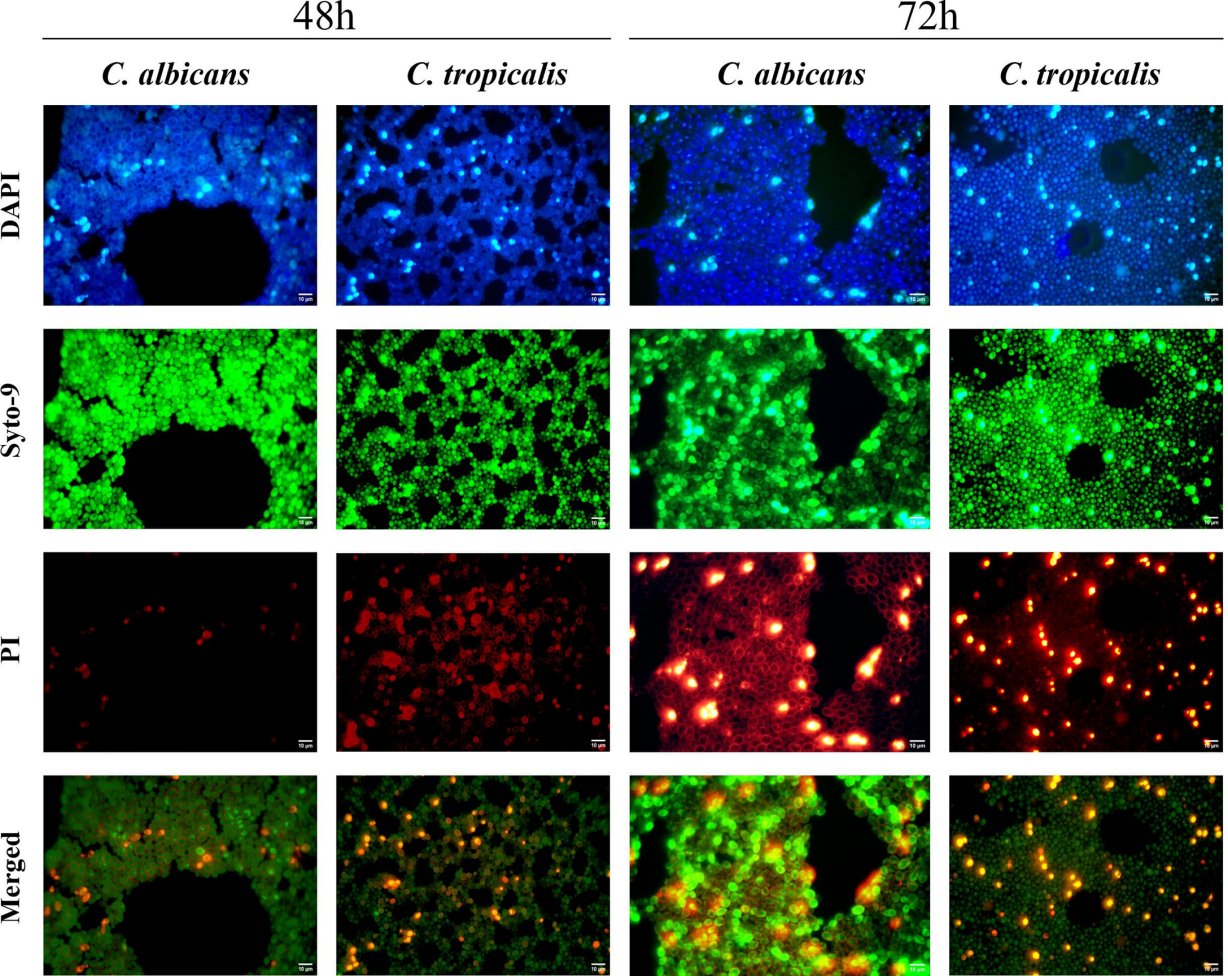
Illustration of the biofilms of *C. albicans* and *C. tropicalis* at 48 and 72 h of growth by epifluorescence microscopy using 4′,6-diamidino-2-phenylindole fluorescent stain and LIVE/DEAD Biofilm Viability Kit. Time samples of 48 and 72 h were used to compare the total cell and live/dead cells in the biofilms using an Olympus BX50 microscope, and pictures were obtained by AmScope software at ×100 magnification. Then, the pictures from each filter were merged in Fiji-ImageJ version 1.57 (Schindelin et al., 2012).

As shown in **Figure 2**, both *Candida* species showed an increase in cells and higher density within the biofilms during the specified time window, although no significant differences were obtained between time samples. Although optimization of the methodology was done during the study, the images of live and dead cells within biofilms of both species did not show the best clarity. However, the merged images evidenced a better clarity of the biofilms, and the macros Biofilms Viability checker allowed us to obtain a trustful evaluation of live and dead cells. No visual differences were detected in the percentage of dead and alive cells between *Candida* species during the specified time window, which was in concordance with the previous statistical analysis, and a homogenous distribution of dead cells within the biofilms of both *Candida* species was shown (see the merged pictures in **Figure 2**). Interestingly, it is possible to observe water channels in both biofilms of *C. albicans* and *C. tropicalis*, wherein a more abundant network of channels was shown in *C. tropicalis* biofilms at 48 h that progressively matured into well-defined channels at 72 h in a similar way as observed in *C. albicans* biofilms at 48 h.

In addition, both *C. albicans* and *C. tropicalis* exhibited mature biofilms with a multilayer growth during the specified time window, which made it difficult to evaluate the average size of cell area in each biofilm and to compare it between the *Candida* species.

**Figure 3:**
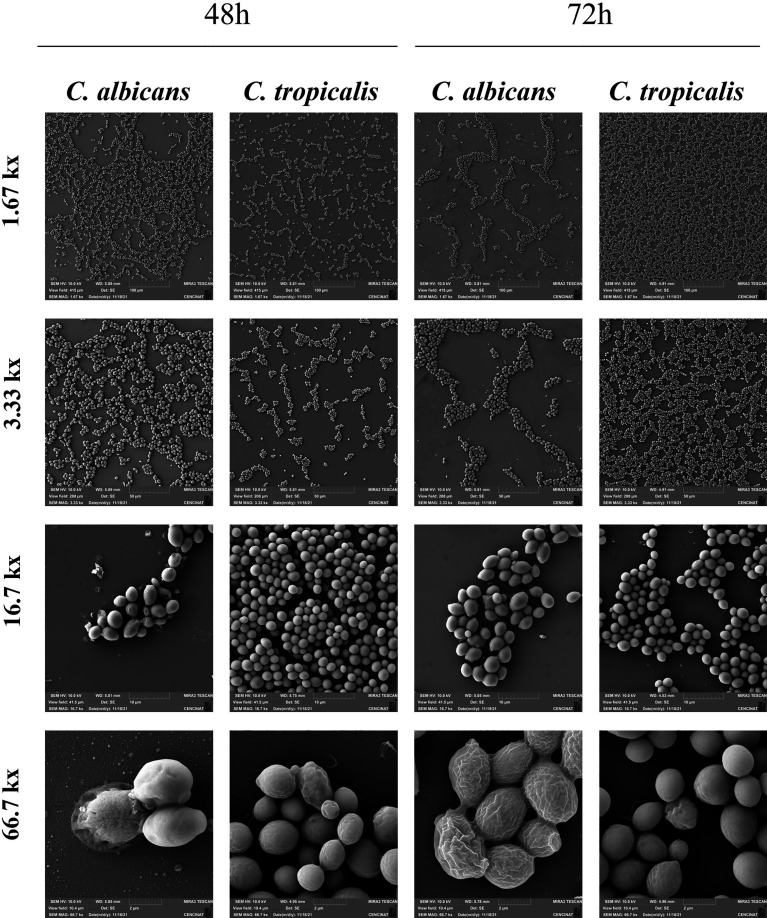
Illustration of the biofilms of *C. albicans* and *C. tropicalis* at 48 and 72 h of growth by scanning electron microscopy using different magnifications (1.67, 3.33, 16.7, and 66.7 kx). Time samples of 48 and 72 h were used to compare the biofilm structure as well as cell morphology and area of the biofilms using a Tescan Mira 3 scanning electron microscope equipped with a Schottky field emission gun (Schottky FEG-SEM). Yeast cell area was calculated from the best pictures by Fiji ImageJ version 1.57 (Schindelin et al., 2012).

As shown in **Figure 3**, the biofilm comparisons between the *Candida* species using SEM analysis were also performed at different magnifications (1.67, 3.33, 16.7, and 66.7 kx). In the lower magnifications, it was possible to observe biofilms with a highly ordered structure of cell assemblages, with multilayer growth, and exhibiting interconnectivity between cell assemblages. At 48 h, both *Candida* biofilms evidenced larger spaces without adhering cells that became shorter in their biofilms of 72 h, thus achieving a mature phase of biofilm. However, at 72 h, the density of cells within the biofilm was notoriously higher in the biofilms of *C. tropicalis*. Meanwhile, at higher magnifications, we were able to analyze the cell areas and morphologies in both biofilms, thus perceiving visible differences in cell area and matrix production between the *Candida* species. As previously indicated in **Table 3**, *C. albicans* showed a significant and superior cell area when compared to *C. tropicalis* biofilm cells in both time samples. A decrease of 12.5% of its mean yeast area was also found for *C. albicans* between 48 and 72 h; meanwhile, for the same time period, an increase of 10% was evidenced for *C. tropicalis.* Moreover, at high magnification (66.7 kx), an irregular texture on the surface of *C. albicans* cells was observed. The frequency of these structures was more prominent at 72 h than at 48 h. This could be caused by the higher production of matrix or extracellular polymeric substance (EPS) during the biofilm stage. However, the *C. tropicalis* biofilm cells did not evidence the same rate of EPS production, at least visually, as those of *C. albicans*.

### 3.5 Comparison of the four methodologies to assess biofilm development

**Figure 4:**
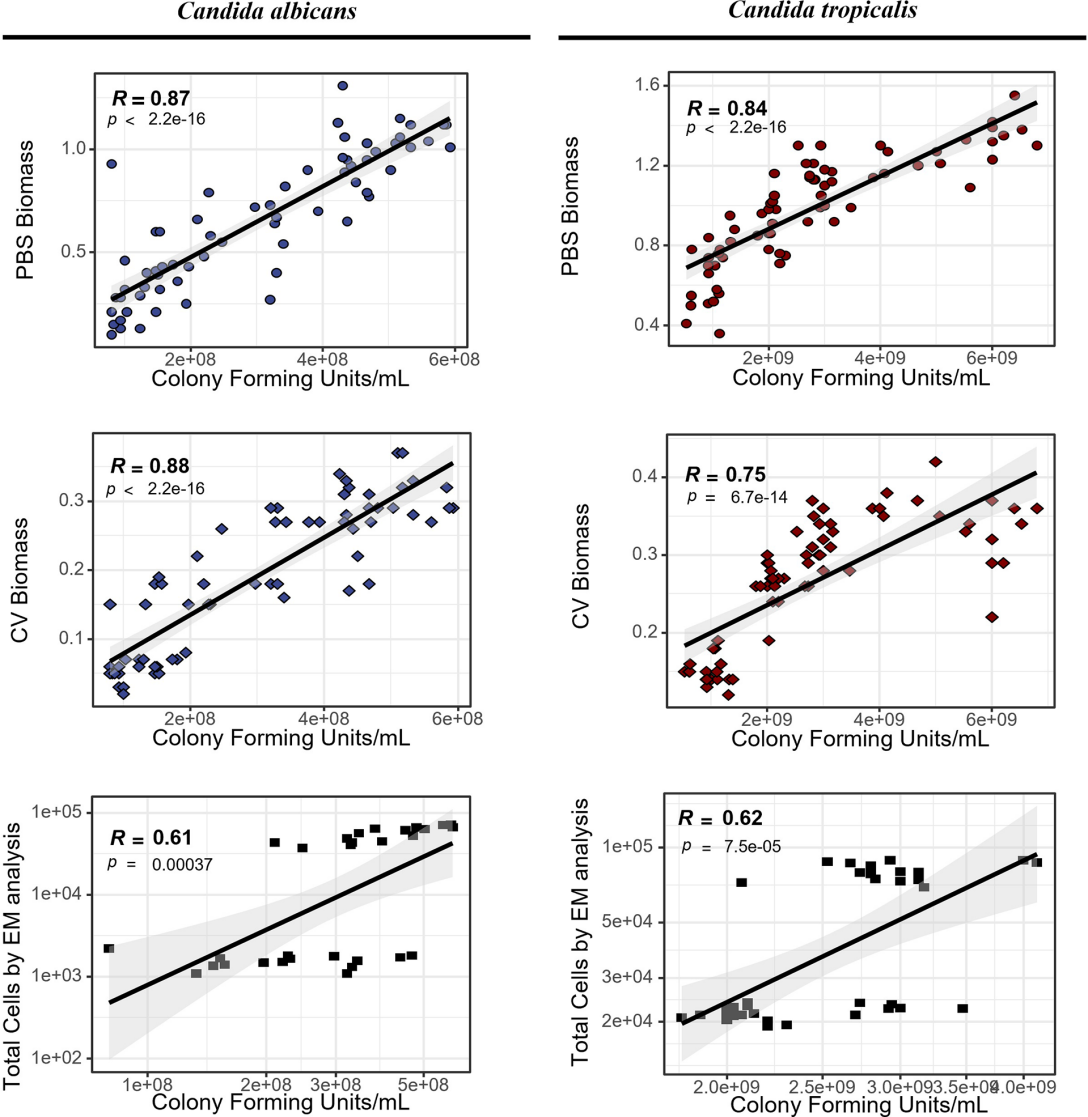
Comparative analysis of biofilm development through crystal violet (CV) staining and phosphate-buffered saline (PBS) suspension for biomass quantification by optical density assays, total cell count by epifluorescence microscopy (EM) analysis, and colony-forming unit (CFU) counting assays. Least-squares linear regression models were used to compare the four methods to assess biofilm development using R studio version 4.0 (RStudio Team, 2021).

After the conclusion of the experimental assays, we compared the most common methods used for biofilm development assessment, which were used in the present study (**Figure 4**). Least-squares linear regression models were applied using CFU counting assays as reference or gold standard methodology, and *R*-squared values were observed as a goodness-of-fit measure for the biofilm analysis.

As expected, the EM analysis evidenced the lowest *R*-squared values for both *Candida*-related biofilms (*R* ^2^ = 0.61–0.62), followed by biomass quantification with CV staining (*R* ^2^ = 0.75–0.88) and then PBS suspension (*R* ^2^ = 0.84–0.87). The extra procedure steps (such as stain, fixation, and washing steps) on CV staining particularly showed a loss of goodness-of-fit measurement on *C. tropicalis* biofilms.

In CV and PBS assays, four-time intervals were analyzed in contrast to two intervals for the EM analysis. Even when each repetition is independently measured, the reduction in the number of points because of the reduced time intervals can affect the statistical estimation and thus impact the comparison with this methodology. Therefore, we re-calculated the *R*-squared as well as the *p*-value restricting all assays for only two time intervals. After these considerations, we found that (1) *C. albicans* showed *R* = 0.66, 0.65, and 0.61 for the PBS, CV, and EM methodologies, respectively, while (2) *C. tropicalis* showed *R* = 0.60, 0.70, and 0.62 for the PBS, CV, and EM methodologies, respectively. In all cases, the *p*-value was inferior to 0.01.

## 4 Discussion

The ability to establish biofilm is considered a main virulence factor in bacterial and fungal infections due to several intrinsic biofilm-associated factors, such as antimicrobial resistance, immune system evasion, and horizontal gene transfer mechanisms in multispecies biofilms (de Barros et al., 2020; Eix and Nett, 2020; Pinto et al., 2021). The formation of *Candida* biofilms has been observed on multiple surfaces, including blood, mucosal surface, and most medical devices (*i*.*e*., nonliving objects in contact with patients’ bodies) (Vitális et al., 2020; Ponde et al., 2021; Pohl, 2022). Both *C. albicans* and non-albicans *Candida* (NAC) species have been found in the developed biofilm stage at several medical devices, such as stents, shunts, implants, endotracheal tubes, pacemakers, and multiple types of catheters (Ponde et al., 2021). Recently, our metanalysis on the prevalence of *Candida* biofilms in bloodstream infections showed that the mortality rate in these infections was 37.9%, of which 70.0% were from biofilm-associated infections (Atiencia-Carrera et al., 2022), showing *C. tropicalis* as the most prevalent species among the biofilm-forming organisms (67.5%), even more than *C. albicans* (30.3%). Therefore, the present study aimed to use multiple methods to compare the biofilm cycle of life between *C. albicans* and *C. tropicalis* and evaluate their ability to establish biofilms. To the authors’ best knowledge, this is the first study to analyze biofilms of these *Candida* species during time by these methodologies as well as evaluate the accuracy in the assessment of biofilm development.

The standard optical density measurement assays offer a quick and relatively high-throughput way to screen microorganisms with the ability for biofilm formation with minimal equipment requirements (Govaert et al., 2019; Fan et al., 2020). In 2022, Castro and colleagues already demonstrated the useful application of biomass assays (such as crystal violet staining) on biofilms, reporting the accuracy in the results obtained in monospecies biofilms, but this method was not able to properly evaluate multispecies biofilms (Castro et al., 2022). The present study showed that, at least for *Candida*-related biofilms, the PBS and CV assays have similar correlations with CFU counting and, in general, are higher than using EM methodology. However, the number of time samples should be increased in future studies to obtain a more reliable statistical analysis.

As expected, both *Candida* species exhibited their ability to form biofilms. Upon evaluation of their biofilms, our results showed a continuous biomass growth until 72 h in both *Candida* species. Our results are in accordance with the typical life cycle described in yeast biofilms (Atriwal et al., 2021), more specifically as follows: (1) attachment and colonization of round yeast cells to a surface, (2) growth and proliferation of yeast cells creating a basal layer of anchoring cells, (3) growth of pseudohyphae (oval yeast cells joined end to end) and hyphae (long cylindrical cells) accompanying the production of extracellular matrix, and eventually (4) dispersal of cells from the biofilm to find new sites to colonize.

Regarding interspecies comparison, it was noted that *C. tropicalis* showed a better ability to form biofilms than *C. albicans*. Our results agreed with the observations from previous studies (Vitális et al., 2020; Konečná et al., 2021). Zuza-Alves *et al*. reported that a biofilm positivity occurred most frequently in the isolates of *C. tropicalis* (Zuza-Alves et al., 2017). Furthermore, Vitális *et al*. demonstrated that all *C. tropicalis* isolates from fatal infections were intermediate/high biofilm producers (Vitális et al., 2020). In 2021, Konečná *et al*. described that *C. tropicalis* could be categorized as a strong biofilm producer due to the biomass production observed in this species (Konečná et al., 2021). The results of the present study are likewise in agreement with our meta-analysis (Atiencia-Carrera et al., 2022). In these studies, *C. tropicalis* was associated with a higher mortality rate when compared with *C. albicans* and other NAC species. This propensity of *C. tropicalis* for dissemination and higher mortality rate could be related to its high biofilm formation as one of the main intrinsic virulence factors exhibited by this species (Zuza-Alves et al., 2017; Kulig et al., 2022). The higher biomass growth of *C. tropicalis* biomass could also provide an advantage to the cells within the biofilm, enabling better protection against antifungal or antimicrobial agents (Sharma et al., 2019).

In the present study, the CFU counting results showed a continuous increment of viable cells over time in the biofilms of both species, but only *C. tropicalis* demonstrated a significant increment of viable cells between 72 and 96 h. Our results on the cycle of life from *C. albicans* biofilms are in agreement with previous studies (Chandra and Mukherjee, 2015; Cavalheiro and Teixeira, 2018; Pohl, 2022) but also demonstrated a longer biofilm cycle of life in *C. tropicalis* species through active cell proliferation within the stationary biomass of the biofilm. Therefore, further studies should be performed to confirm the extension of the *C. tropicalis* biofilm cycle of life (such as the dispersal of cells from the biofilm) and to evaluate the survival rate of cells when exposed to several antifungal treatments by themselves or combined with alternative compounds as recently realized by Galdiero et al. in *C. albicans* biofilms (Galdiero et al., 2020).

Furthermore, *C. tropicalis* biofilms exhibited a continuously and statistically higher CFU counting when compared with *C. albicans*. These results corroborated recent studies proposing the classification of *C. tropicalis* as a strong biofilm producer (Konečná et al., 2021) and its association with a higher mortality rate in hospitalized patients (Vitális et al., 2020). The infectivity of yeasts depends on specific virulence mechanisms that confer the ability to colonize host surfaces, invade deeper host tissue, or evade host defenses (Eix and Nett, 2020). *C. tropicalis*’ ability to produce steady biofilms demonstrated an important clinical impact once biofilm-associated infections are currently difficult to treat, representing a serious source of reinfections (Zuza-Alves et al., 2017; Marak and Dhanashree, 2018).

The EM and SEM findings confirmed the higher biofilm production of *C. tropicalis* when compared with *C. albicans*. *C. tropicalis* also demonstrated a higher number of total cells within biofilms through DAPI staining. The EM analysis with DAPI is a useful and cheap methodology that allows total cell count within biofilms as previously described in studies by Castro and colleagues (2022; Castro et al., 2021). Meanwhile, live/dead staining provided information on how many of the total cells were dead and alive within biofilms through their capacity to exclude, accumulate, and metabolize the fluorophores Syto-9 and PI (Rosenberg et al., 2019; McGoverin et al., 2020). Therefore, live/dead staining was the most variable of the methodologies used in this work. As expected, no statistical differences were found in the percentage of dead and alive cells within the biofilm between *C. albicans* and *C. tropicalis*. The reason for the absence of a statistical difference could be that a greater biofilm growth in *C. tropicalis* was reached but it maintained a similar proportion between dead and alive cells when compared to *C. albicans* biofilm, although the amount of dead and alive cells within *C. tropicalis* biofilm was statistically significant. Therefore, further studies with metabolic assays and gene expression/genetic analysis should be conducted to demonstrate similar and divergent phenotypic expression and its relationship with these *Candida* species’ metabolism. Another explanation could be the intrinsic variability of live/dead staining with Syto-9 and PI, so further studies should be performed to minimize the previously cited limitations and optimize the resolution of this methodology.

It is well known that biofilms formed by different *Candida* species may vary in morphology and density, showing a polymeric extracellular matrix that protects the biofilm cells and water channels, as previously described in bacterial biofilms (2019; Handorf et al., 2018). The extracellular matrix components also differ from those found in the *Candida* cell wall, and these moieties are proposed to modulate host recognition by concealing the cell wall that typically interacts with the immune system (de Barros et al., 2020; Eix and Nett, 2020). The EM analysis also evidenced water channels within the biofilms of *C. tropicalis* that progressively matured until 72 h in well-defined channels as reported in *C. albicans* biofilms at approximately 38–72 h (Cavalheiro and Teixeira, 2018) and *Staphylococcus aureus* biofilms at 24 h (Konduri et al., 2021). In the SEM analysis, both *Candida* species strongly adhered to the abiotic surfaces (glass slide) and then subsequently developed into a mature biofilm within 48 h. However, when assessing the cell morphologies between these *Candida* species, *C. albicans* demonstrated a superior cell area when compared to *C. tropicalis* cells. Another visual difference was the irregular texture on the surface of *C. albicans* biofilm cells indicating a higher production of EPS when compared to *C. tropicalis* at 72 h, which is in agreement with the literature (Corte et al., 2019; Pohl, 2022). Although *C. albicans* visually showed a higher amount of EPS, both *Candida* species evidenced a confluent basal blastospore layer covered by a matrix of EPS and a few hyphal elements, similar to the findings described by Cavalheiro and Teixeira (2018). These hyphal elements are believed to play an important role in fungal infection as previously described in *C. albicans* (Gulati et al., 2018; Lohse et al., 2018), being also identified in biofilms of *C. tropicalis* in the present study.

Some authors reported that *Candida* biofilms begin to disintegrate at 72 h (Seneviratne et al., 2009; Rodríguez-Cerdeira et al., 2020); however, we observed mature biofilms without signs of disintegration and a low number of dead cells within the biofilm. Concerning cell morphologies, cell differentiation to opposite mating types and switching from yeast to filamentous form (hyphae or pseudohyphae) are examples of individual yeast cell differentiation. Both processes have been investigated using different yeast species, and they can contribute to the virulence and invasiveness of pathogenic *Candida* species (Alves et al., 2020; Ponde et al., 2021; Pohl, 2022). In this study, it was only possible to analyze the adhering cells that formed a multilayer consortium in the biofilm, and therefore the lack of yeast germination (pseudohyphae/hyphae forms) constituted one limitation of the present work that should be rectified in further studies by optimizing several factors (such as pH conditions and carbon sources). The characterization of *C. tropicalis* biofilms is currently an important research field due to the emerging cause of hospital-acquired infections worldwide (Alkharashi et al., 2019; Zhang et al., 2020; Atiencia-Carrera et al., 2022; Kulig et al., 2022).

However, the present study has additional shortcomings, such as the absence of analyses based on metabolic or gene expression, flow cytometry, confocal microscopy, and quantitative polymerase chain reaction to assess the differences between *C. albicans* and *C. tropicalis* biofilms, and only one specimen of each *Candida* species was evaluated, although each strain belonged to a reference microbial collection culture (ATCC and INSPI).

## 5 Conclusions

The present study demonstrated the ability of *C. tropicalis* to produce a strong biofilm by comparing its biofilm cycle of life with *C. albicans*. To the authors’ best knowledge, this is the first study to simultaneously analyze the biofilms of these *Candida* species during the specified time window (until 96 h) by biomass assays (crystal violet staining and PBS suspension), colony-forming unit counting, epifluorescence microscopy, and scanning electron microscopy. Our results evidenced a higher biomass growth, viable cell production, and total cell count in *C. tropicalis* biofilms. Water channels in biofilms *C. tropicalis* progressively matured in well-defined channels at 72 h as observed in *C. albicans* biofilms at 48 h. Meanwhile, *C. albicans* biofilms showed a superior cell area and higher matrix production. Finally, when evaluating the applied methodologies, the PBS suspension was shown to be similar to CV staining for biomass quantification by optical density assays. From our results, new questions about the physiology of these biofilms and the forces that modulate yeast behavior remain unanswered; therefore, further studies should analyze the gene expression and metabolic network that influence the evolution of the biofilm formed by different *Candida* species.

## Data availability statement

The original contributions presented in the study are included in the article/**Supplementary Material**. Further inquiries can be directed to the corresponding authors.

## Author contributions

Experimental research: MA-C, FC-M, and KV. Methodology: AD and AM. Validation: AD, ET, and AM. Formal analysis: MA-C, FC-M, KV, AD, ET, and AM. Resources: AD and AM. Data curation: AD, ET, and AM. Writing—original draft preparation: MA-C and AM. Writing—review and editing: AD, ET, and AM. Supervision: AM. Project administration and funding: AM with USFQ Chancellor Grants. All authors contributed to the article and approved the submitted version.

## Funding

This work was supported by the COCIBA research budget from Universidad San Francisco de Quito, under project ID 12260 entitled “Adhesión inicial y resistencia antimicrobiana de *Candida* sp. aisladas de la microbiota humana” and project ID 16801 entitled “Characterization of single and mixed biofilms”. The funders had no role in the study design, data collection and analysis, decision to publish, or preparation of the manuscript. The APC was funded by the Research Office of Universidad San Francisco de Quito.

## Acknowledgments

All colleagues at the Microbiology Institute of USFQ, COCIBA, and Research Office of Universidad San Francisco de Quito deserve special recognition for their support in this study. In particular, we are grateful to David Valencia and Darío Cueva for their assistance in epifluorescence microscopy assays.

## Conflict of interest

The authors declare that the research was conducted in the absence of any commercial or financial relationships that could be construed as a potential conflict of interest.

## Publisher’s note

All claims expressed in this article are solely those of the authors and do not necessarily represent those of their affiliated organizations, or those of the publisher, the editors and the reviewers. Any product that may be evaluated in this article, or claim that may be made by its manufacturer, is not guaranteed or endorsed by the publisher.

## Supplementary material

Supplementary Material Information S1 Summary of materials and methods used to grow *Candida* biofilms and their further analysis illustrating the normality assessment study (**Supplementary Table S1**) and a brief description of the procedure for image counting and related programs.

Supplementary File S2 Excel file with crude data and cured process analysis.

Supplementary File S3 Excel file with data on total cell counting through epifluorescence microscopy analysis.
