# Supplementary material for: Evaluation of the biofilm life cycle between Candida albicans and Candida tropicalis
Source: Front Cell Infect Microbiol. 2022 Aug 18;12:953168. doi: 10.3389/fcimb.2022.953168 (PMC9433541; doi:10.3389/fcimb.2022.953168)
Supplement: Supplementary Material Information S1 — Summary of materials and methods used to grow Candida biofilms and their further analysis illustrating the normality assessment study ( Supplementary Table S1 ) and a brief description of the procedure for image counting and related programs. [file DataSheet_1.docx]

# **S.1 Supplementary Material.**


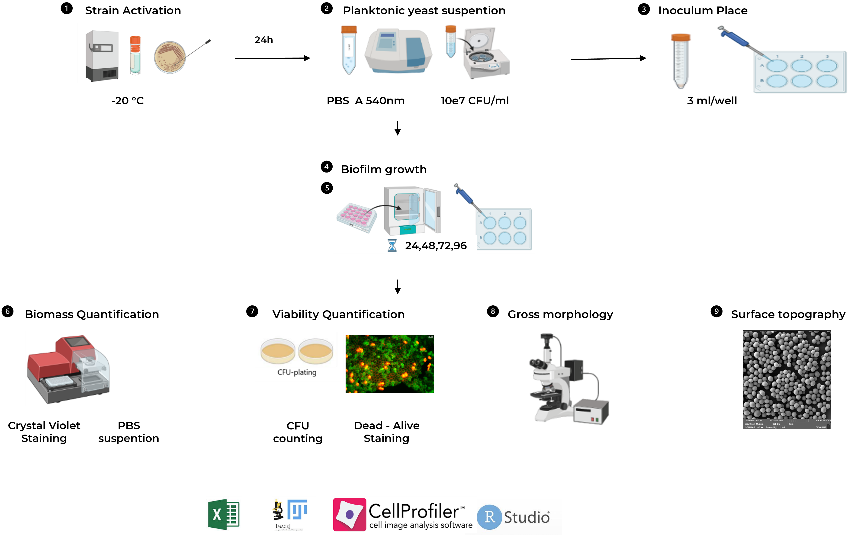


Supplementary Figure S1: Summary of material and methods used to grow Candida species biofilms and analysis of them.

## **S.2 Normality assessment**


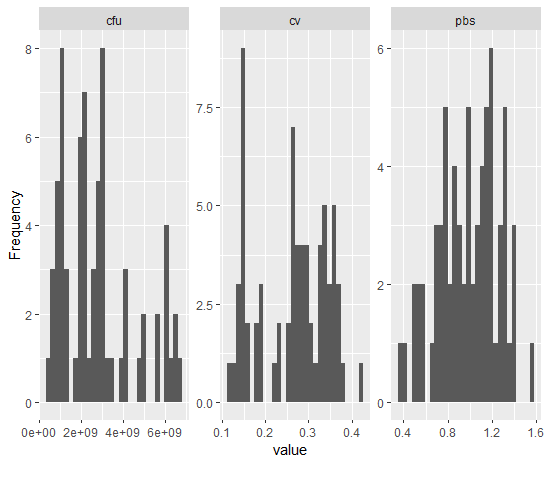

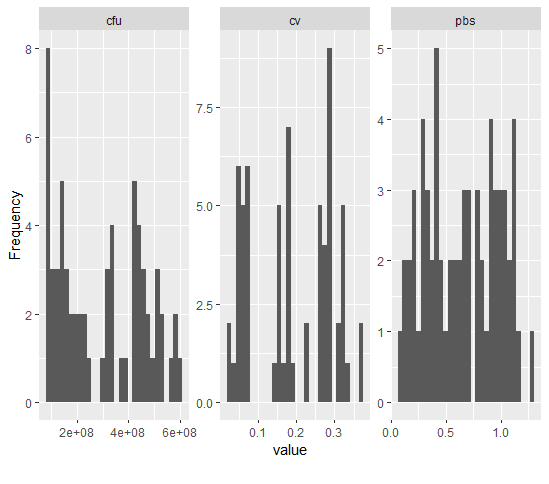


**a**

**b**

Supplementary Figure S2: Histograms of total data for two species of Candida. Each histogram displays the distribution of data values for continue variables (tree assays). Vertical axis represents the count (frequency), and the horizontal axis represents the possible range of the data values. This information was used in conjunction whit Shapiro Wilk results and qq-plot to evaluate normality in data.


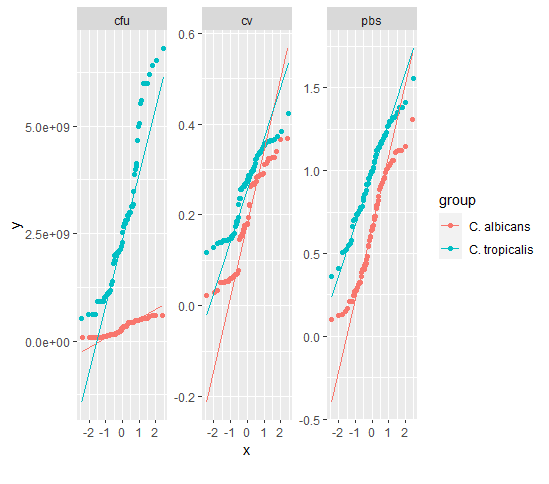


Supplementary Figure S3: quantile-quantile plots for data distribution of Candida spp. biofilms growth divide by specie. Plots shows non normality distribution and could be beneficial transform CFU scale in logarithmical scale.

Note: The function will take a long time with many observations, so you may choose to specify an appropriate

## **S.3 Supplementary Table S1.** Comparison intraspecies of biomass and viability assays by time points through Kruskal Wallis nonparametric test, followed by Dunn’s test using a Benjamini–Hochberg adjustment for multiple comparisons.

| **Species** | **Type of assays** | **Kruskal Wallis test**  ***p-value*** | **group1** | **group2** | **n1** | **n2** | **Benjamini–Hochberg p-adjustment method**  ***p-value*** |
| --- | --- | --- | --- | --- | --- | --- | --- |
| *Candida albicans* | PBS Biomass | 4.33E-11 | 24 | 48 | 20 | 15 | 1.62E-02 |
|  |  |  | 24 | 72 | 20 | 15 | 5.55E-06 |
|  |  |  | 24 | 96 | 20 | 15 | 7.63E-11 |
|  |  |  | 48 | 72 | 15 | 15 | 4.55E-02 |
|  |  |  | 48 | 96 | 15 | 15 | 1.57E-04 |
|  |  |  | 72 | 96 | 15 | 15 | 6.10E-02 |
|  | CV  Biomass | 2.19E-12 | 24 | 48 | 20 | 15 | 1.01E-02 |
|  |  |  | 24 | 72 | 20 | 15 | 2.06E-07 |
|  |  |  | 24 | 96 | 20 | 15 | 1.59E-11 |
|  |  |  | 48 | 72 | 15 | 15 | 1.44E-02 |
|  |  |  | 48 | 96 | 15 | 15 | 1.23E-04 |
|  |  |  | 72 | 96 | 15 | 15 | 1.34E-01 |
|  | CFU  viability | 4.59E-10 | 24 | 48 | 20 | 15 | 1.55E-02 |
|  |  |  | 24 | 72 | 20 | 15 | 6.59E-07 |
|  |  |  | 24 | 96 | 20 | 15 | 6.13E-09 |
|  |  |  | 48 | 72 | 15 | 15 | 1.55E-02 |
|  |  |  | 48 | 96 | 15 | 15 | 1.42E-03 |
|  |  |  | 72 | 96 | 15 | 15 | 3.87E-01 |
| *Candida tropicalis* | PBS Biomass | 6.10E-12 | 24 | 48 | 20 | 15 | 7.04E-03 |
|  |  |  | 24 | 72 | 20 | 15 | 2.83E-07 |
|  |  |  | 24 | 96 | 20 | 15 | 6.50E-11 |
|  |  |  | 48 | 72 | 15 | 15 | 7.04E-03 |
|  |  |  | 48 | 96 | 15 | 15 | 5.05E-05 |
|  |  |  | 72 | 96 | 15 | 15 | 1.73E-01 |
|  | CV  Biomass | 1.58E-11 | 24 | 48 | 20 | 15 | 5.61E-04 |
|  |  |  | 24 | 72 | 20 | 15 | 8.69E-09 |
|  |  |  | 24 | 96 | 20 | 15 | 1.18E-09 |
|  |  |  | 48 | 72 | 15 | 15 | 1.21E-02 |
|  |  |  | 48 | 96 | 15 | 15 | 4.04E-03 |
|  |  |  | 72 | 96 | 15 | 15 | 6.90E-01 |
|  | CFU  viability | 4.20E-13 | 24 | 48 | 20 | 15 | 7.05E-04 |
|  |  |  | 24 | 72 | 20 | 15 | 2.28E-06 |
|  |  |  | 24 | 96 | 20 | 15 | 3.14E-13 |
|  |  |  | 48 | 72 | 15 | 15 | 8.77E-02 |
|  |  |  | 48 | 96 | 15 | 15 | 3.60E-05 |
|  |  |  | 72 | 96 | 15 | 15 | 1.89E-02 |

# **S.4 Counting Supplementary Information**

DAPI photographs for each time point (12 per day ,3 different days) was analyzed whit the pipeline to obtain total cells per image, the average and SD was then calculated.

The pipeline used to execute the total counting of yeast was constructed in Cell Profiler following the tutorials available in the web site <https://cellprofiler.org>.

The procedure is detailed in the following images.


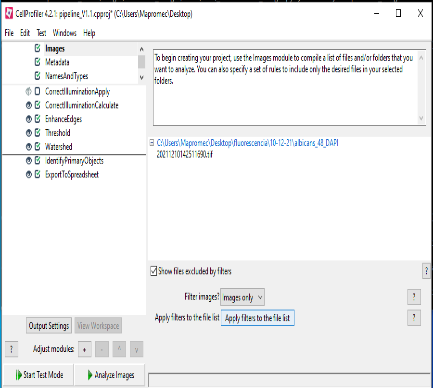


1.- Charge the images dragging into the space designed by default.


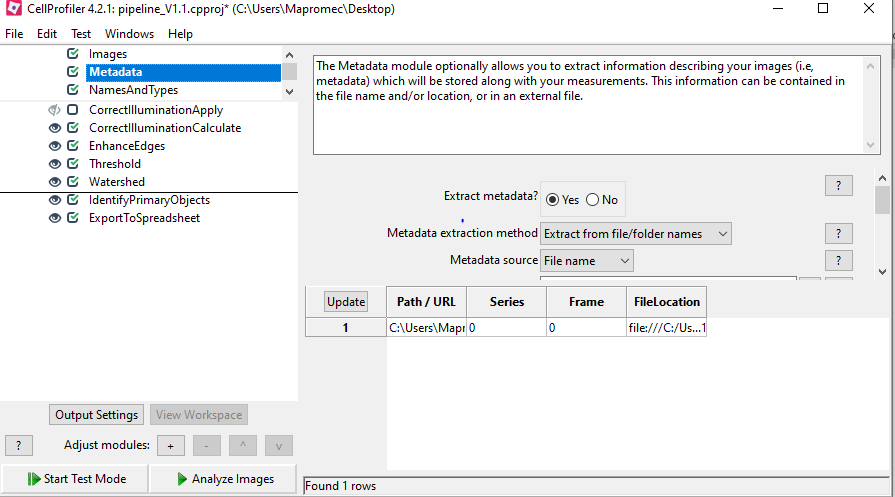


2.- Allow the software access to metadata of the images


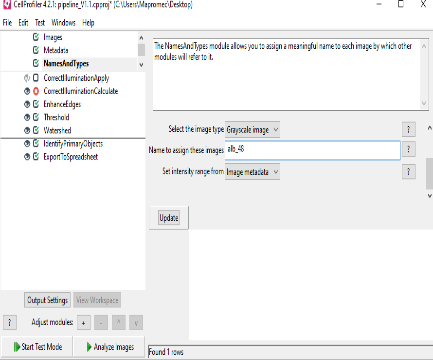


3.- Assign one name for all your bundle of pictures

After step 3 pipeline will execute without problems, the process will depend on the ram memory of your computer.

## **Result of counting**


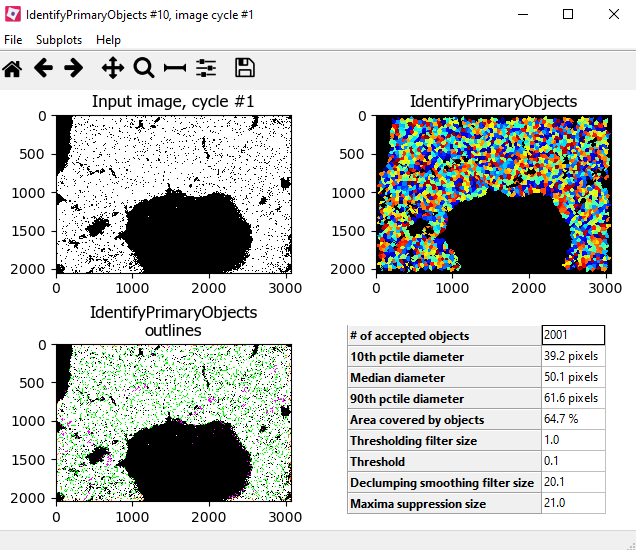

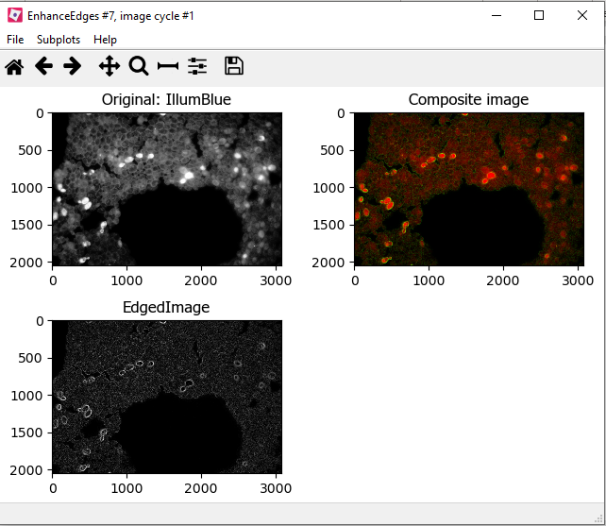


Red arrows show the counting for this image, in our case we process 12 images in bundle, so we use the terminal and see the results in a excel sheet at the final of process.

## **Dead-Alive process.**

To dead alive we follow the process exactly as describe in the paper *“Biofilm viability checker: An open-source tool for automated biofilm viability analysis from confocal microscopy images*” [12]. The following image is an example of the overlayed image output from the macro, as well as the results of the analysis of the sample image*.*


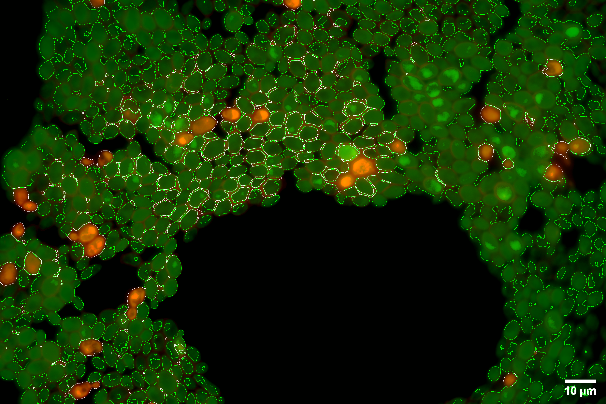


Macros Result. This image is generated automatically by the program and save in the chose location


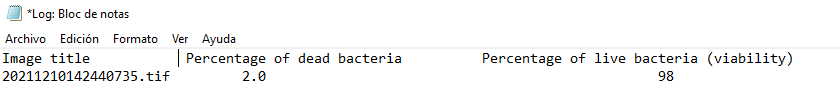


Result generate automatically and save in the same place of the image above.
